# Real-Time Patient and Staff Radiation Dose Monitoring in IR Practice

**DOI:** 10.1007/s00270-016-1526-8

**Published:** 2016-12-09

**Authors:** Anna M. Sailer, Leonie Paulis, Laura Vergoossen, Axel O. Kovac, Geert Wijnhoven, Geert Willem H. Schurink, Barend Mees, Marco Das, Joachim E. Wildberger, Michiel W. de Haan, Cécile R. L. P. N. Jeukens

**Affiliations:** 1 Department of Radiology, Maastricht University Medical Centre, P. Debyelaan 25, 6229 HX Maastricht, The Netherlands; 2 Department of Radiology, Stanford University Medical Centre, 300 Pasteur Drive, R354, Stanford, CA 94303 USA; 3 Department of Vascular Surgery, Maastricht University Medical Centre, Debyelaan 25, 6229 HX Maastricht, The Netherlands; 4 CARIM School of Cardiovascular Diseases, Maastricht University Medical Centre, 6229 HX Maastricht, The Netherlands

**Keywords:** Endovascular procedures, Radiation exposure, Radiation dosimetry, Patient dose, Radiation monitoring, Interventional radiology, Radiation protection

## Abstract

**Purpose:**

Knowledge of medical radiation exposure permits application of radiation protection principles. In our center, the first dedicated real-time, automated patient and staff dose monitoring system (DoseWise Portal, Philips Healthcare) was installed. Aim of this study was to obtain insight in the procedural and occupational doses.

**Materials and Methods:**

All interventional radiologists, vascular surgeons, and technicians wore personal dose meters (PDMs, DoseAware, Philips Healthcare). The dose monitoring system simultaneously registered for each procedure dose-related data as the dose area product (DAP) and effective staff dose (E) from PDMs. Use and type of shielding were recorded separately. All procedures were analyzed according to procedure type; these included among others cerebral interventions (*n* = 112), iliac and/or caval venous recanalization procedures (*n* = 68), endovascular aortic repair procedures (*n* = 63), biliary duct interventions (*n* = 58), and percutaneous gastrostomy procedure (*n* = 28).

**Results:**

Median (±IQR) DAP doses ranged from 2.0 (0.8–3.1) (percutaneous gastrostomy) to 84 (53–147) Gy cm^2^ (aortic repair procedures). Median (±IQR) first operator doses ranged from 1.6 (1.1–5.0) μSv to 33.4 (12.1–125.0) for these procedures, respectively. The relative exposure, determined as first operator dose normalized to procedural DAP, ranged from 1.9 in biliary interventions to 0.1 μSv/Gy cm^2^ in cerebral interventions, indicating large variation in staff dose per unit DAP among the procedure types.

**Conclusion:**

Real-time dose monitoring was able to identify the types of interventions with either an absolute or relatively high staff dose, and may allow for specific optimization of radiation protection.

## Introduction

The number and complexity of vascular and non-vascular fluoroscopy-guided interventions continuously increased and have led to an increased radiation exposure for interventional radiologists, surgeons, and supporting medical staff members [1–4]. Radiation safety in fluoroscopy-guided interventions is crucial for patient care quality assurance as well as for occupational safety. Occupational radiation exposure results predominantly from scattered radiation originating from the patient toward the medical staff [5]. Levels of procedural radiation exposure are affected by multiple factors and many are beyond operator control, e.g., the type and complexity of the performed procedure or the dimensions of the patient within the X-ray field of view. Other factors can be at least partially controlled, such as the position of the medical staff relative to the patient, the X-ray equipment and acquisition technique (fluoroscopy, digital subtraction angiography (DSA), roadmap, or 3D), and the radiation protection tools used. Detailed knowledge of the radiation exposure during specific fluoroscopy-guided procedures, thereby optimizing the layout of the angio-suites, should be an integral part of the development of X-ray systems and interventional techniques in order to reduce exposure for both staff and patients [6].

Several studies have evaluated either the patient or occupational radiation exposure during fluoroscopy-guided interventions, such as endovascular aortic repair (EVAR) procedures and cardiologic interventions [7–10]. However, studies presenting comprehensive data on the combined patient and staff dose are lacking due to the absence of dose monitoring systems that can efficiently and accurately co-register patient and staff dose. Given the need of comprehensive dose analysis in interventional radiology practice, the aim of the current study was to implement a comprehensive procedural and occupational real-time dose monitoring system to obtain insight in the procedural and occupational dose for a wide variety of procedures. This might provide valuable and detailed insights in differences in radiation exposure between patients and staff in various types of procedures, based on which suggestions can be made to improve working habits and radiation safety.

## Materials and Methods

A dedicated real-time dose monitoring system (DoseWise Portal, Philips Healthcare, Best, the Netherlands) was installed in our angio-suite and hybrid operating room (Allura Xper with ClarityIQ, Philips Healthcare, Best, the Netherlands). This system automatically and simultaneously registered patient and staff dose for each procedure by combining (1) radiation dose structured reports from the X-ray system that contained all the system performance data such as the dose area product (DAP) and acquisition type (fluoroscopy, DSA, roadmap, or 3D imaging) with (2) real-time personal dose meters (PDM, DoseAware, Philips Healthcare, Best, the Netherlands) worn by all the staff members.

Each medical staff member (physicians and radiology technicians) was equipped with a PDM attached to the left breast pocket outside their protective lead apron. The PDMs were calibrated to measure the personal dose equivalent Hp(10) [Sv], which served as an estimator for the effective dose, E [Sv] [11]. For reference, a PDM was mounted on the C-arm at an angle of 45°, under the table when the C-arm was in posterior–anterior position. This reference PDM recorded the scattered dose at a fixed distance from the iso-center without any additional shielding [2]. Use and type of additional in-room radiation shielding as well as the presence and role of the staff members during each procedure were recorded. To analyze staff and patient exposure on both procedural and single X-ray event level, an in-house software program was written (Mathematica Version 10.2, Wolfram Research Inc., Champaign, IL). This software performed a co-registration of the radiation doses for the patient (reported as DAP), first operator (FO), and first radiology technician (FT), with influencing factors such as procedure type, acquisition techniques, and use of radiation shielding tools.

In this prospective study, all procedures performed in our angio-suite and hybrid operating room between October 2015 and June 2016 were consecutively included. Procedures were grouped by procedure type, which was based on both treated body part and position of the first operator. The ten most performed procedure types were analyzed and included in the results of the current study (*n* = 587). The study pool consisted of a total of 112 cerebral procedures, 82 visceral and renal artery interventions, 68 iliac and/or central venous chronic obstruction recanalization procedures, 63 EVAR procedures, 62 AV fistula procedures, 58 biliary interventions, 54 pelvic arterial interventions, 32 percutaneous nephrostomy procedures, 28 superficial femoral and/or crural artery interventions, and 28 percutaneous gastrostomy procedures (Table 1). Fluoroscopy X-ray was used in low-dose mode and only occasionally switched to medium or high dose when necessary, according to the standard clinical practice at our institution. Real-time, in-room qualitative feedback on their current dose rate (dose/second, in color-coding) was provided to medical staff 24 month prior to the start of the study and was continued during the study.

**Table 1:** Distribution of procedural DAP and medical staff doses for different groups of interventions

		Cerebral interventions	AV fistula interventions	Percutaneous gastrostomy	Biliary interventions	Visceral and renal a. interventions	Endovascular aortic repair	Percutaneous nephrostomy	Iliac artery interventions	Venous recanalization	Femoral and crural a. interventions
DAP (Gycm²)											
Median		29.8	3.5	2.0	8.7	79.9	84.2	5.0	31.9	47.6	13.2
25% Quartile		17.6	2.0	0.8	3.4	40.4	52.5	1.5	19.2	16.9	6.6
75% Quartile		44.5	5.5	3.1	16.9	145.3	147.0	8.3	59.9	97.3	20.7
Min		5.2	0.2	0.4	0.3	0.9	16.8	0.3	4.1	1.1	0.7
Max		211.5	25.5	12.4	69.0	365.2	931.9	211.5	289.4	399.4	52.4
*n*		112	62	28	58	82	63	32	54	68	28
FO dose (μSv)											
Median		3.2	1.9	1.6	13.7	29.9	33.4	3.1	19.7	17.4	12.8
25% Quartile		0.8	0.9	1.1	3.0	10.8	12.1	0.6	6.1	5.0	3.7
75% Quartile		12.2	5.4	5.0	43.0	91.2	125.0	12.0	45.4	33.7	30.1
Min		<0.1	<0.1	<0.1	<0.1	0.7	1.2	<0.1	0.2	<0.1	<0.1
Max		55.3	64.4	47.4	118.0	603.9	614.0	38.0	274.1	172.9	45.8
*n*		71	48	21	47	69	60	19	45	52	16
FT dose (μSv)											
Median		1.4	1.8	1.4	2.8	4.4	3.5	1.6	2.9	2.6	1.3
25% Quartile		0.6	0.4	0.3	0.7	1.2	0.9	0.4	0.8	1.4	0.4
75% Quartile		3.6	4.1	4.4	6.9	7.8	11.4	5.5	6.0	8.3	3.0
Min		<0.1	0.1	<0.1	<0.1	<0.1	<0.1	<0.1	<0.1	<0.1	<0.1
Max		75.8	19.4	11.1	51.2	136.7	547.0	11.7	32.3	124.6	75.3
*n*		82	49	22	39	63	50	17	45	51	20

*a* Arterial, *SD* standard deviation, *FO* first operator, *FT* first technician

### Statistical Analysis

Quantitative data were tested for normal distribution and were displayed as median and interquartile range (IQR) where applicable. Correlations between personal doses and procedure DAP were examined using linear regression. Association between type of radiation shielding and relative FO and FT doses were analyzed by means of cross-tabulation analysis. (SPSS statistics 21.0, Chicago, Illinois). *P* values <0.05 were considered significant.

## Results

### Patient and Medical Staff Doses

**Fig. 1:**
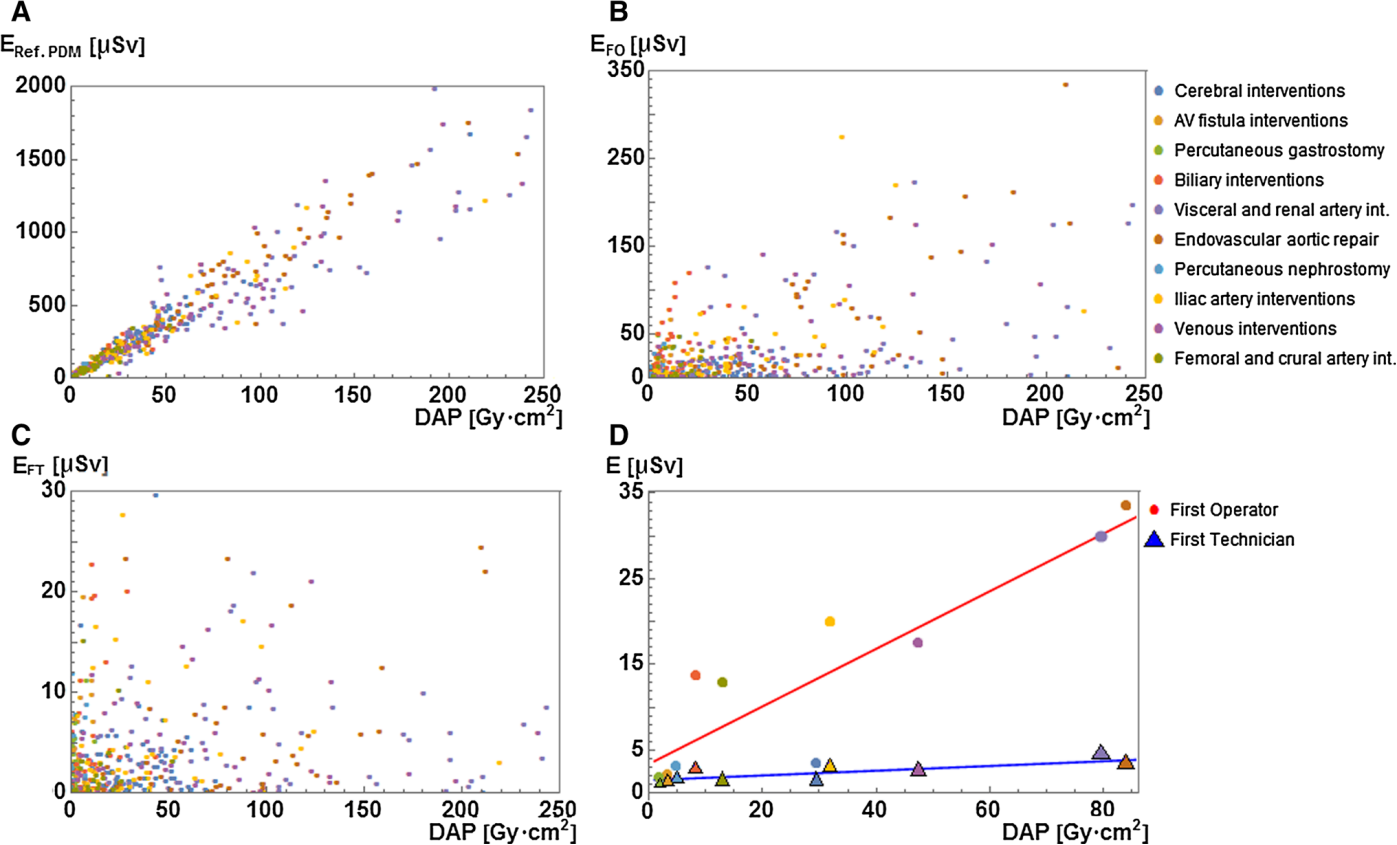
**A** Correlation between the effective dose from the reference PDM and the procedural DAP for all procedures. Coefficient of correlation *R* ^2^ = 0.94. **B** Correlation between effective first operator dose (*E*_FO_) and procedural DAP for all the procedures. *R* ^2^ = 0.37. **C** Correlation between effective first technician dose (*E*_FT_) and procedural DAP for all procedures. *R* ^2^ = 0.07. **D** Correlation between median *E*_FO_ and median *E*_FT_ with median procedural DAP. *R* ^2^ (FO) = 0.8; *R* ^2^ (FT) = 0.66

In Fig. 1, the correlation between the effective dose of (A) the reference PDM (C-arm) (*n* = 587), (B) the FO (*n* = 440), and (C) the FT (*n* = 415) with the procedural DAP is shown for all procedures independent of the type of the procedure. There was a strong correlation between procedural DAP and reference PDM dose (*R* ^2^ = 0.94) and a weak correlation between procedural DAP and FO dose or FT dose (*R* ^2^ = 0.37, and *R* ^2^ = 0.07, respectively). The correlation between median FO and FT doses and median DAP for the ten specified procedure types was strong (Fig. 1D, *R* ^2^ = 0.80, slope = 0.34 μSv/Gy cm^2^; and *R* ^2^ = 0.66, slope = 0.028 μSv/Gy cm^2^, *p* < 0.001 and *p* = 0.004, respectively).

**Fig. 2:**
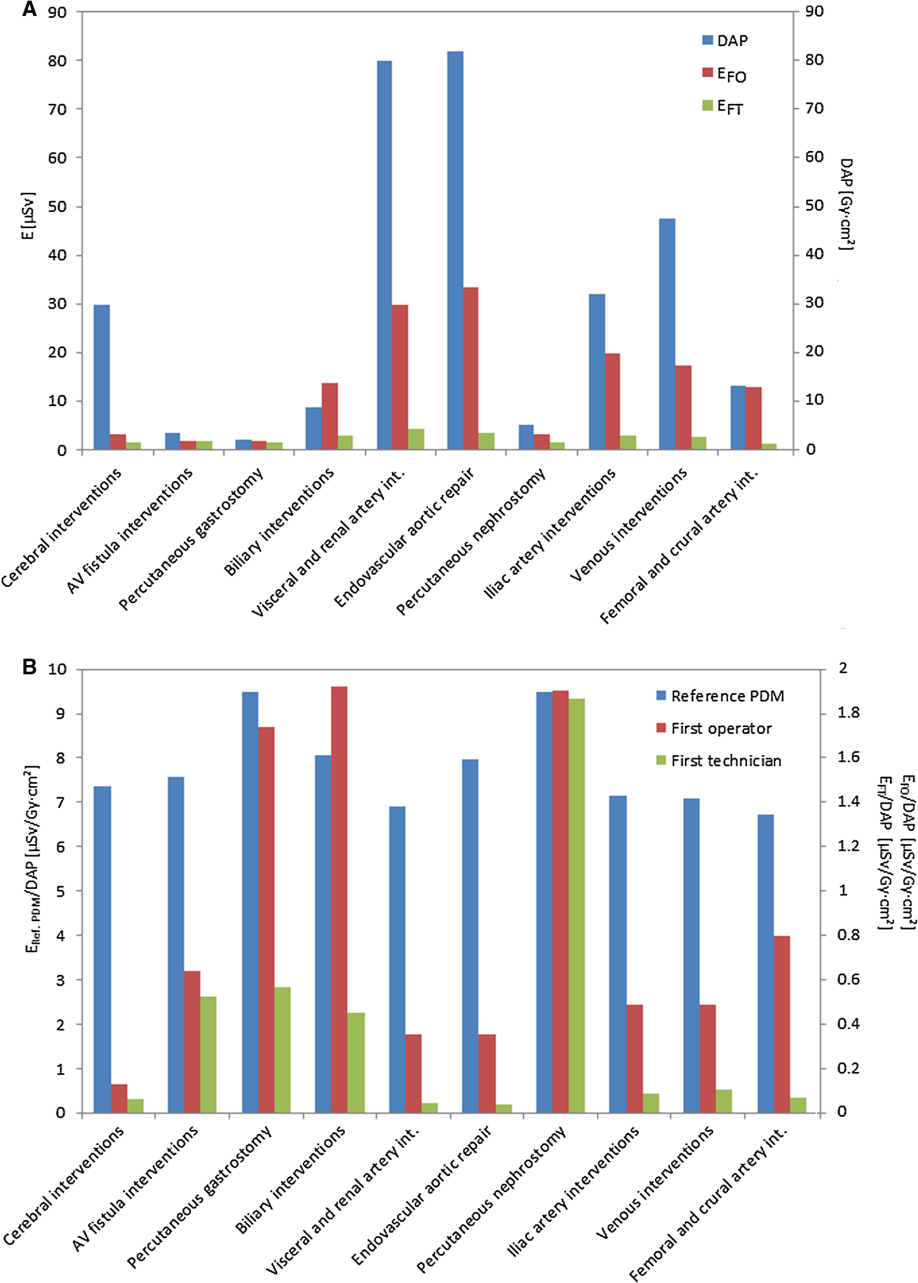
Effective doses (*E*) obtained from the FO PDMs and the FT PDMs (*left axis*) and procedural DAP (*right axis*). *E*_FO_ and *E*_FT_ values in absolute numbers (**A**) and normalized to procedural DAP (**B**). *Bars* represent the median effective dose for each procedure type

Figure 2A shows that the median procedural and staff doses vary widely between procedure types (see also Table 1 for more detailed information). Median (±IQR) DAP was highest for EVAR procedures (84.2 Gy cm^2^ [52.5–147.0 Gy cm^2^]) and visceral and renal arterial interventions (79.9 Gy cm^2^ [40.4–145.3 Gy cm^2^]), and lowest for AV fistula procedures (3.5 Gy cm^2^ [2.0–5.5 Gy cm2]) and percutaneous gastrostomy procedures (2.0 Gy cm^2^ [0.8–3.1 Gy cm^2^]). FO dose was highest for aortic and visceral procedures (33.4 μSv [12.1–125.0 μSv] and 29.9 μSv [10.8–91.2 μSv], respectively), and lowest for AV fistula maintenance and percutaneous gastrostomy (1.9 μSv [0.9–5.4 μSv] and 1.6 μSv [1.1–5.0 μSv], respectively). Median FT doses were highest for abdominal and pelvic venous and arterial procedures, but were <5.0 μSv for all procedure types.

When FO and FT doses were normalized to (corrected for) procedural DAP, the relative dose displayed a different distribution compared to the absolute dose, namely procedure types having a high relative dose, e.g., biliary interventions as well as nephrostomies and gastrostomy interventions and a low relative dose, e.g., cerebral interventions (Fig. 2B). The normalized reference PDM dose was, as expected, relatively constant between procedures.

### Acquisition Techniques

**Fig. 3:**
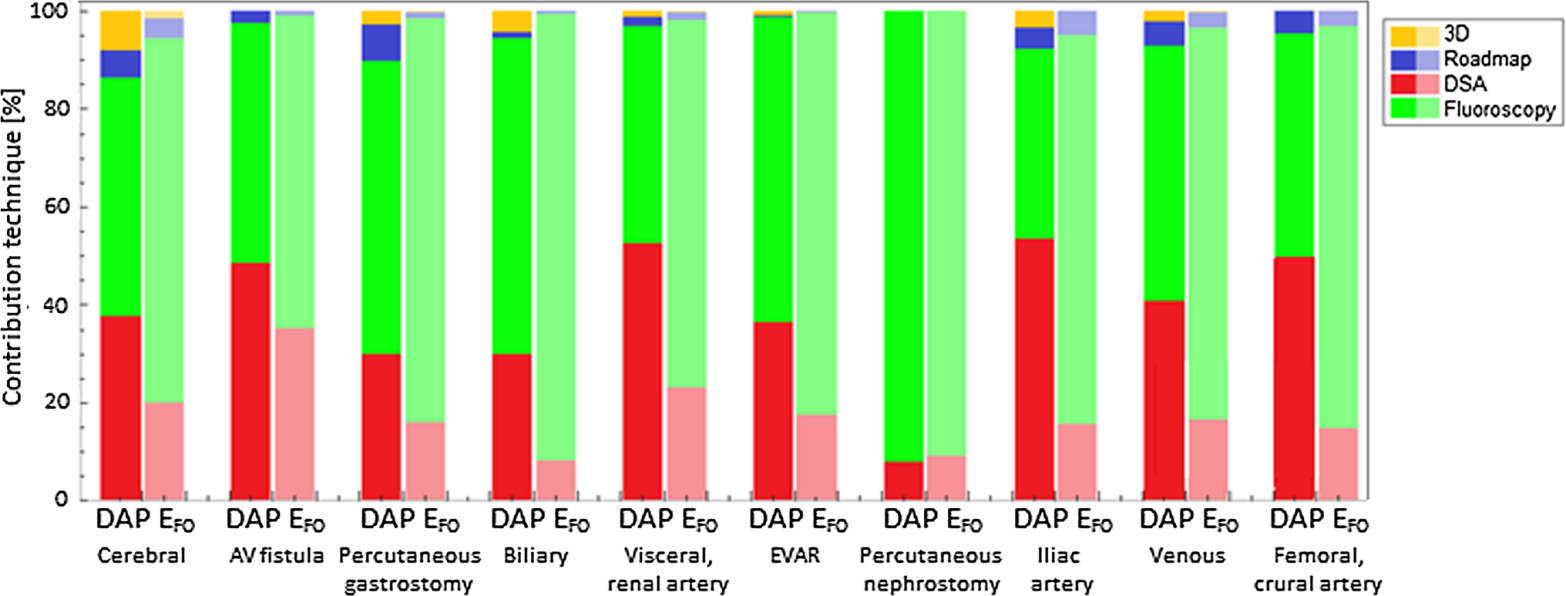
Contribution of fluoroscopy, DSA, roadmap, and 3D acquisitions to total procedural DAP and to the effective dose of the first operator (*E*_FO_). Relative dose values are shown for different procedure types

The relative and absolute contributions of different acquisition techniques to procedural DAP and FO doses are graphically displayed in Fig. 3A, B respectively. Procedural (patient) dose was strongly driven by both, fluoroscopy and DSA, (mean 56 and 39%, respectively), whereas fluoroscopy was the main contributor to FO dose for all procedure types (mean 80%), thereby indicating that in most cases, the staff left the angio-suite or kept large distance to the C-arm during the acquisition of DSA. Roadmapping and 3D acquisitions had little contribution to both absolute and relative patient and staff doses.

### In-Room Shielding

Use of additional in-room shielding differed between procedure types, as shown in Table 2a. Table side shielding was used more often than ceiling-mounted shielding. In EVAR procedures, no in-room shielding was used due to specific set-up of the hybrid operating room. Among all individual procedures, independent from the procedure type, non-significant but lower median FO and FT doses were observed when using ceiling-mounted or table side shielding (Table 2b).

**Table 2:** (a) Use of in-room shielding per procedure group, (b) Crosstabs of relative FO and FT dose (E_FO_/DAP and E_FT_/DAP) with and without ceiling-mounted shielding

(a)	Table-side shield use (%)	Leaded ceiling-suspendedshield use (%)	*N*
Cerebral interventions	68	49	112
AV fistula interventions	48	32	62
Percutaneous gastrostomy	75	36	28
Biliary interventions	64	40	58
Visceral and renal artery int.	71	52	82
Endovascular aortic repair	0	0	63
Percutaneous nephrostomy	43	26	32
Iliac artery interventions	60	53	54
Venous interventions	48	30	68
Femoral and crural artery int.	61	54	28

(b) *E*_FO_/DAP (*n* = 440)	Without	With	*p* value
Table-side shield	1.11 ± 1.67 µSv/Gy cm² (*n* = 171)	1.10 ± 1.62 µSv/Gy cm² (*n* = 269)	*p* = 0.95
Ceiling-suspended shield	1.17 ± 1.85 µSv/Gy cm² (*n* = 258)	1.01 ± 1.31 µSv/Gy cm² (182)	*p* = 0.32

*E*_FT_/DAP (*n* = 415)	Without	With	
Table-side shield	0.57 ± 1.45 µSv/Gy cm² (*n* = 155)	0.460 ± 1.16 µSv/Gy cm² (*n* = 260)	*p* = 0.39
Ceiling-suspended shield	0.57 ± 1.37 µSv/Gy cm² (*n* = 234)	0.41 ± 1.13 µSv/Gy cm² (181)	*p* = 0.19

## Discussion

Ionizing radiation used for medical purposes carries the risk of radiation-induced tissue reactions and stochastic effects for both patients and medical staff [12]. While patient dose is justified by medical indication, radiation exposure for healthcare professionals has to be monitored carefully due to its repetitive character and potential long-term effects. It is clear that the medical staff working with ionizing radiation should be aware of the radiation dose they may receive during a particular procedure and which factors determine the level of these doses [13]. Knowledge on personal and procedural radiation dose allows for optimal use of the ALARA principles [6, 13]. As such, dose monitoring systems could be recommended as an integral part of the clinical workflow.

The present study provides comprehensive patient and staff dose analysis for various types of interventional radiology procedures. The results show that absolute and relative exposure to medical staff strongly depends on the type of procedure performed. A categorization of procedure types based on 1) treated body part and 2) position of the physicians seems a useful categorization as it reflects the differences in scatter from the patient’s body parts (e.g., abdominal versus cerebral interventions) as well as differences in distance of the physician to the X-ray source, (e.g., biliary versus vascular abdominal interventions). Such a categorization enabled structural analysis of occupational dose with respect to optimization of radiation protection and furthermore will allow for simplified comparison and adaption of results between centers if broadly adopted. Absolute doses of the first operators were lowest for cerebral interventions and highest for EVAR and visceral abdominal interventions, with the median doses varying up to a factor 10. This may be explained by differences in procedure complexity, distance between the scatter source and the first operator, and the use of shielding. After normalizing to procedural DAP, the relative first operator doses were highest for biliary interventions and percutaneous nephrostomy, indicating that with the current procedure set-up and available radiation protection tools as available in our angio-suites, radiologists were not able to protect themselves sufficiently from the scatter radiation during these interventions [14, 15]. Especially in these procedures with high absolute and relative doses, efforts should be made for optimization of radiation protection measures.

The median first technicians’ (FT) doses were below 5 µSv for all procedure types, which is up to factor 13 lower than the median first operator (FO) doses. This difference reflects the fact that technicians have more possibilities to maximize the distance to the patient and thus minimize the in-room exposure. However, for AV fistula interventions and percutaneous nephrostomy, the median FT dose was almost equal to the median FO dose, which may be explained by the fact that in these procedures, FT often stand close to the patient to either control the X-ray panel or comfort the patient. Moreover, individual FT doses were as high as >500 μSv in particular procedures.

The existing knowledge on the cancer risk from medical staff exposure levels is mainly based on the epidemiologic life span study analysis by extrapolation to the low-dose regime (<50 mSv) [16, 17]. Such extrapolations may likely introduce a bias in risk assessment, especially for very low exposure levels [12]. A recently published prospective study [18] on more than 90,000 radiology technicians observed an elevated risk of brain cancer, breast cancer, and melanoma among technicians who work with ionizing radiation compared to those who never did. In particular, the risk of lethal brain cancer was 2.55 times increased, and the risk of breast cancer and melanoma were elevated 1.3 and 1.16 times in technicians assisting in fluoroscopy-guided interventions. Another recently published study [19] compared the mortality rates between 43,763 radiologists and 64,990 psychiatrists in the US. The authors found an excess risk of acute myeloid leukemia and/or myelodysplastic syndrome mortality in radiologists who graduated before 1940 likely due to occupational radiation exposure as well as an increased mortality risk for melanoma, non-Hodgkin lymphoma, and cerebrovascular disease. The authors found no evidence of increased mortality among radiologists who graduated more recently (not analyzed by subspecialty). Both studies lack personal radiation exposure data, so cancer risk could not be linked to individual exposure levels. Considering these studies, although the medical staff exposure per procedure from our study might seem reasonably low, efforts to apply ALARA principles should be taken very seriously for both technicians and physicians.

For some types of interventions, radiation protection tools were not available or could not be used. This may be partly due to individual neglect of physicians and technologists during the procedure, but also due to potentially avoidable barriers, for example our angio-suite design (ceiling shield has a fixed working distance) or system design (lead shields applicable on side of the table only, lead drapes not applicable at all) as well as sterile working conditions interfering with current radiation protection tools. There was a trend toward a reduced staff dose when using ceiling-mounted shielding. This result however was not significant which may be explained by the fact that in our current clinical practice, shielding is often only applied after the start of a procedure, thereby missing the initial X-ray events and leading to suboptimal radiation shielding. Furthermore, as the PDMs were attached on the level of the breast, the effect of using table lead drapes on the staff dose cannot be elucidated due to this location.

With respect to exposure from different types of X-ray acquisitions, we found that procedural (patient) dose was mainly driven by DSA, while staff dose originated predominantly from fluoroscopy. This indicates that DSA was a clearly avoidable form of exposure to the medical staff and proved that all staff members should ideally leave the angio-suite with DSA being performed from the control room or shield or maximize the distance to the X-ray source whenever possible. Remarkably, the contribution of roadmap acquisitions to total procedural and staff dose was low for all procedure types. Roadmaps require contrast agent injection by hand and thus presence of the first operator in the angio-suite. Further analysis has to be performed on patient and staff radiation reduction potential using roadmaps instead of DSA with respect to the desired image quality.

There are limitations to this study. Firstly, electronic PDMs were not worn by the medical staff during all procedures. First operator PDMs were registered in 440 out of 587 procedures and FT PDMs in 415 procedures, respectively. For relative FO and FT analysis, consequently, only corresponding DAP data were used for procedures in which a staff PDM was registered. Secondly, real-time live feedback on the current dose rate was provided to the medical staff 24 months prior to the start of the study and during the study. The provided data therefore might reflect exposure based on a prior learning curve and general higher awareness for occupational exposure. In clinics without real-time feedback, actual exposure might therefore be higher and a larger dose reduction may be feasible after introducing real-time monitoring and personal feedback. Thirdly, the presented exposure levels reflected the procedure types most often performed at our institution. This certainly does not cover the full repertoire of interventional radiology procedures. Future research in other centers has to be performed to provide exposure levels for more procedure types and other angio-suites. This could ultimately help to develop benchmark values for patient and staff exposure.

In conclusion, comprehensive monitoring of patient and staff dose is crucial for continuous optimization of radiation safety. This study provides expectable exposure levels for medical staff from certain types of interventional procedures and offers suggestions for optimizing radiation protection. The benefit for Patients from minimally invasive interventional procedures is immense and indisputable. However, medical staff and health care providers must continue their efforts to keep radiation exposure as low as reasonably achievable for both patients and staff. Individual dosimetry is a necessary and unquestionable part of this effort.
